# Hypertriglyceridemia Induced Pancreatitis Due to Brentuximab Therapy: First Case Report

**DOI:** 10.7759/cureus.5138

**Published:** 2019-07-15

**Authors:** Sandhya Maradana, Padmastuti Akella, Swarna S Nalluru, Vishal Jindal, Ahmad D Siddiqui

**Affiliations:** 1 Internal Medicine, St. Vincent Hospital, Worcester, USA; 2 Hematology and Oncology, St. Vincent Hospital, Worcester, USA

**Keywords:** brentuximab, acute pancreatitis, hypertriglyceridemia, t cell lymphoma, lipid disorders, adverse drug reactions

## Abstract

Brentuximab vedotin is used for relapsed classical Hodgkin’s lymphoma and mature T-cell lymphomas. We present a unique case of severe hypertriglyceridemia after one dose of single-agent brentuximab therapy. A Middle-Eastern male with a history of primary progressive cutaneous gamma/delta T-cell lymphoma was started on single-agent brentuximab vedotin therapy. Two weeks after single dose brentuximab therapy, he complained of severe epigastric pain, nausea, vomiting and was admitted to the intensive care unit with acute pancreatitis. Physical examination revealed an acutely ill patient with abdominal tenderness and laboratory data showed triglyceride levels of 3175 mg/dL, glycated hemoglobin (HbA1C) 9%, lipase 145 U/L and glucose 594 mg/dL. Computed tomography scan of the abdomen and pelvis confirmed acute interstitial pancreatitis. With medical management patient triglyceride levels decreased and the patient improved. This is the first case report in literature depicting, brentuximab induced hypertriglyceridemia leading to acute pancreatitis. It is a serious complication and can be lethal. Therefore, it is critical to maintain a high index of suspicion for hypertriglyceridemia induced pancreatitis after single dose brentuximab therapy.

## Introduction

Brentuximab vedotin (Adcetris® Seattle Genetics Inc., Bothell, US) is an antibody-drug conjugate (ADC) comprising of a CD30-directed antibody conjugated to a microtubule-disrupting agent, monomethyl auristatin E (MMAE) via a protease cleavable linker [1,2]. Studies have shown that single-agent brentuximab vedotin (BV) treatment can induce an objective response in relapsed T-cell lymphoma with CD30 expression [3]. Effects of BV are dose-dependent and mostly include peripheral neuropathy and neutropenia [2]. Only nine cases of acute pancreatitis have been reported with this drug in literature [4,5]. No reports of brentuximab induced dyslipidemia/hypertriglyceridemia causing acute pancreatitis have been reported. We aim to establish an important adverse effect of this single-agent therapy to raise awareness regarding the clinical safety of brentuximab single-agent therapy.

## Case presentation

A 38-year-old male with a history of gastroesophageal reflux disease, attention deficit disorder and depression was diagnosed with primary progressive cutaneous gamma/delta T-cell lymphoma after he underwent an excisional biopsy of a subcutaneous nodule in his abdomen. His positron emission tomography (PET) scan at the time of diagnosis showed involvement of numerous subcutaneous soft tissue densities in the neck, abdomen, bilateral upper and lower extremities. Additional lesions were also seen along the abdomen, flanks and the gluteal region. Inguinal and common femoral lymphadenopathy was also described at the time of diagnosis. He is a former smoker, denies alcohol consumption and has a family history of hypertension. His only home medications are omeprazole and methylphenidate. For the first three months after diagnosis, he received three cycles of CHOEP (cyclophosphamide, doxorubicin, vincristine, etoposide, prednisone). Due to the progression of the disease, he was treated with four cycles of gemcitabine, oxaliplatin and high-dose dexamethasone therapy for the next three months. He did not pursue follow-up for one year. He returned in one year with fevers, night sweats, marked fatigue and worsening abdominal pain from subcutaneous nodules. His re-staging PET scan showed interval increase in the number and the metabolic activity of innumerable cutaneous lesions in the chest, abdomen, pelvis and a new nodule in the submental region. Given his progression of the disease, he went on to receive six cycles of romidepsin. Being refractory to three lines of therapy thus far and continuing to express CD30+ on a portion of his tumor cells, he began single-agent brentuximab therapy in late last year, with the aim to undergo non-myeloablative allogeneic stem transplant once disease control was achieved.

**Figure 1:**
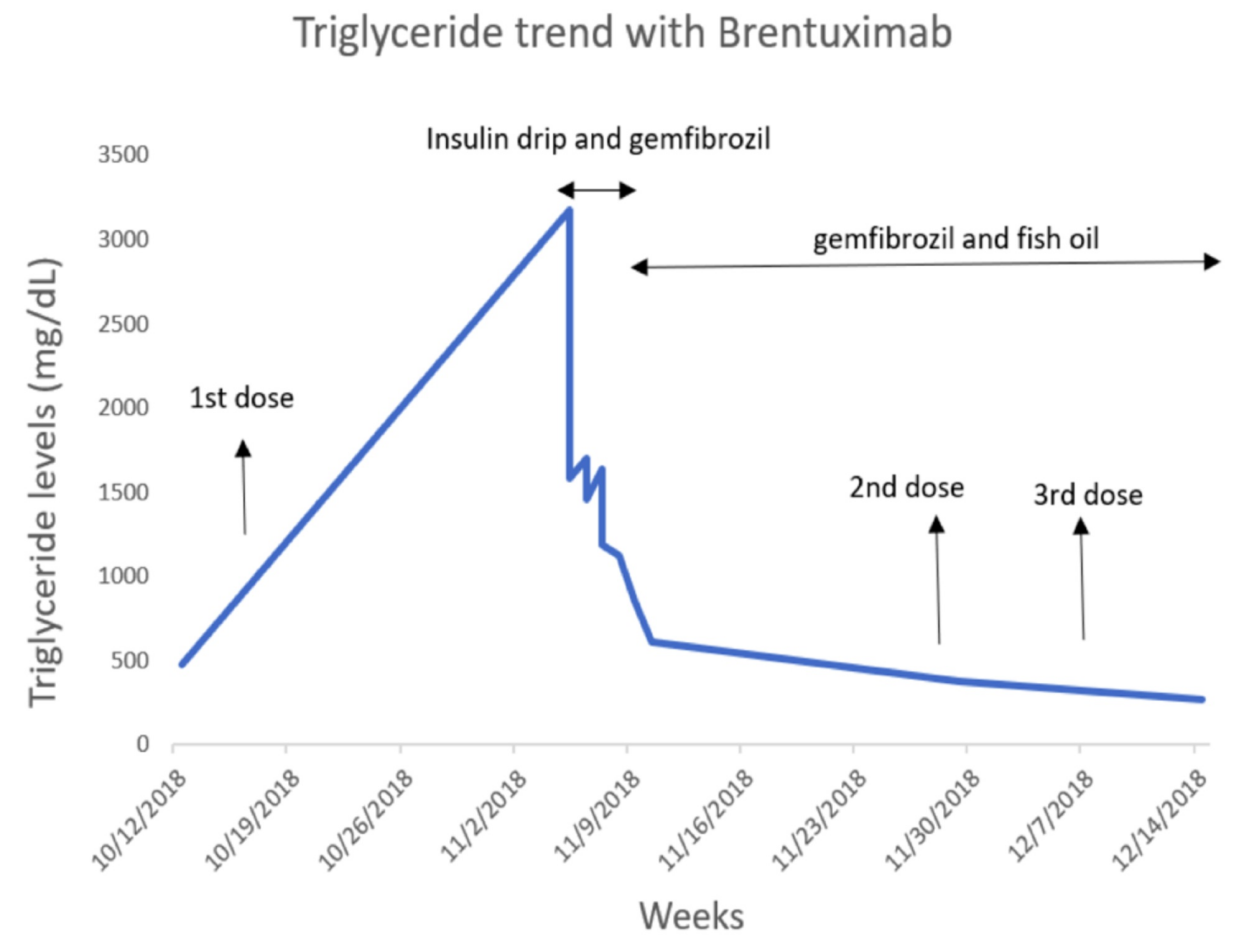
Triglyceride trend with brentuximab Triglyceride trend prior to the start of brentuximab therapy, during the acute intensive care hospitalization, levels after beginning fibrate therapy and restarting single-agent brentuximab therapy.

**Figure 2:**
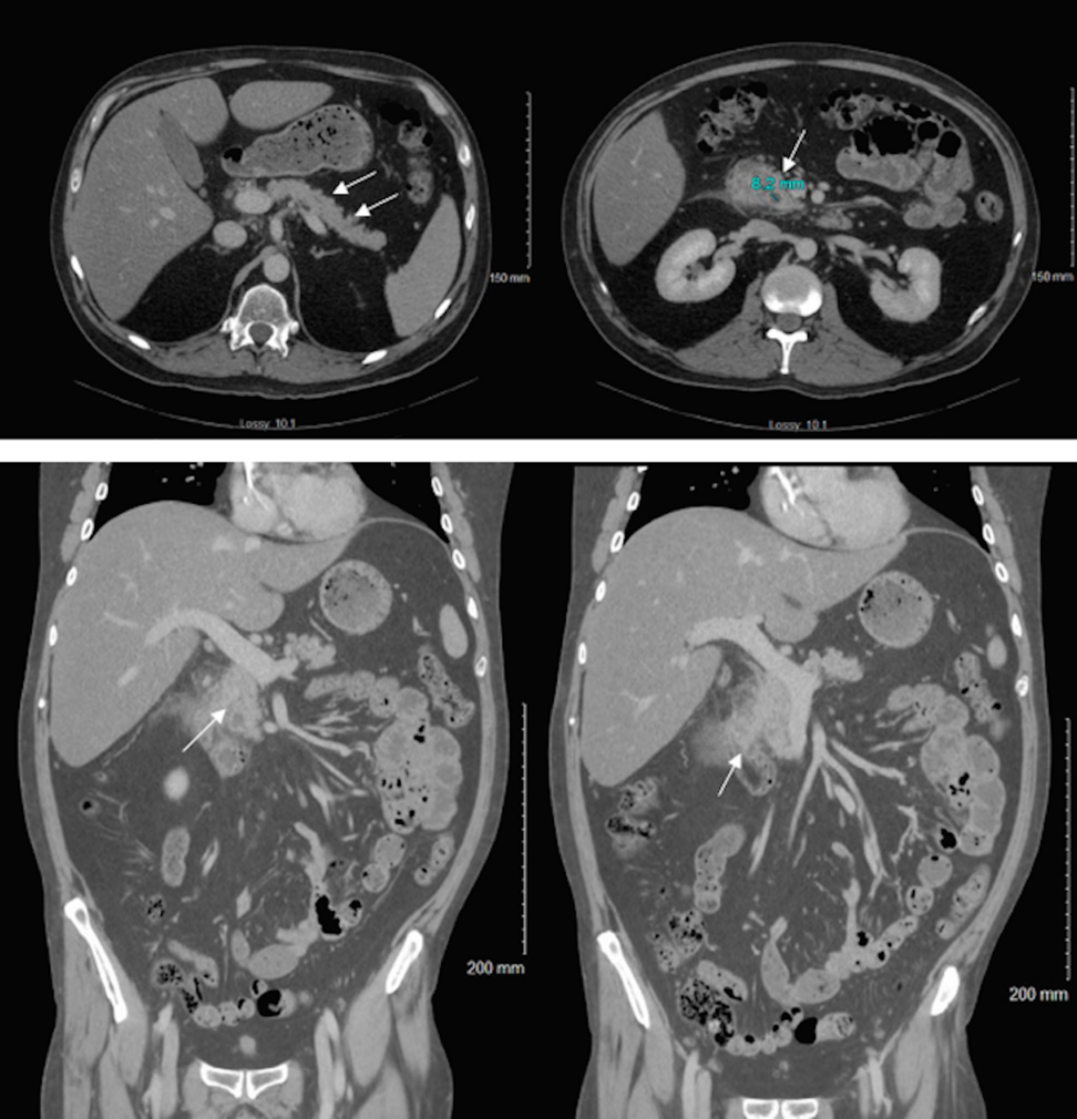
Abdominal computed tomographic (CT) images of acute interstitial pancreatitis Abdominal computed tomography without contrast showing diffuse swelling (arrows, top left) of the pancreas with fat stranding and an 8.2mm fluid collection within the pancreatic head likely pseudocyst (arrow, top right). Coronal sections (arrows, bottom right and left) consistent with acute interstitial pancreatitis but no pancreatic duct stone or ductal dilatation. Duodenal thickening is seen around the head of the pancreas (bottom right).

Two weeks after single-agent brentuximab vedotin therapy, he complained of severe epigastric pain, nausea, vomiting and was admitted to our intensive care unit with acute pancreatitis. Physical examination revealed an acutely ill, dehydrated patient. On presentation, his body temperature was 97.5°F, heart rate was 89 beats per min, blood pressure was 140/97mmHg, and the patient was saturating at 96% on room air. Pink conjunctiva, bilateral adequate breath sounds, soft however mild right upper quadrant and left upper quadrant abdominal tenderness with normal active bowel sounds were noted. His laboratory data of peripheral blood showed a leukocyte count of 5,300/mm3, hemoglobin 13.3 g/dL and platelet count 115000/mm3. His creatinine 0.85mg/dL, total bilirubin 0.2mg/dL and ceruloplasmin levels were 20.6mg/dL. At the time of admission, triglyceride levels were 3175 mg/dL (Figure 1), glycated hemoglobin (HbA1C) 9%, lipase 145 U/L, aspartate aminotransferase 43 U/L, alanine aminotransferase 158 U/L, alkaline phosphatase 138 U/L, lactic acid 3.0 mmol/L, glucose 594 mg/dL, immunoglobulin G 602 mg/dL, immunoglobulin G1 166 mg/dL, immunoglobulin G2 202 mg/dL, immunoglobulin G3 29 mg/dL, and immunoglobulin G4 25 mg/dL. In addition, his anti-smooth muscle antibody, hepatitis C antibody and fourth-generation HIV screen were negative. Patient’s Ranson’s Criteria score on admission was one point. Computed tomography (CT) scan of the abdomen and pelvis showed acute interstitial pancreatitis and hepatic steatosis with no gallstones (Figure 2). He received aggressive fluid resuscitation, pain control with morphine, and was started on intravenous insulin. As his abdominal symptoms improved, he was started on gemfibrozil 600 milligrams twice daily along with fish oil capsule 2000 milligrams twice daily. Triglycerides down trended to 610 mg/dL within five days and lipase normalized within three days. He was resumed on his next cycle of brentuximab at the same dose but was delayed by a week due to the hospitalization. Repeat triglyceride levels after two further doses of brentuximab with the above-mentioned fibrate therapy and dietary changes showed stable triglyceride levels.

## Discussion

Cutaneous gamma delta T-cell lymphoma is a rare form of the T-cell lymphoproliferative disease that represents <1% of primary cutaneous lymphomas and typically exhibits an aggressive phenotype. Brentuximab vedotin is an anti-CD30 monoclonal antibody, which combined with cytotoxic agent monomethyl auristatin E (MMAE) has become a standard and effective therapy for patients with relapsed or refractory CD30+ T-cell lymphomas [6]. As previously seen, abdominal pain has been reported in up to 18% of patients treated with brentuximab [5]. The ALCANZA trial (Trial of Brentuximab Vedotin (SGN-35) Versus Physician's Choice (Methotrexate or Bexarotene) in Patients With CD30-Positive Cutaneous T-Cell Lymphoma) reported a two-percent incidence of hypertriglyceridemia of any grade with brentuximab but no grade 3 or 4 events were reported [7]. It also reported that brentuximab can cause nausea (36%), fatigue (29%), vomiting (17%) and diarrhea (29%) in patients. Pancreatitis was a previously unidentified severe adverse effect in phase 3 ALCANZA trial but there have been several cases of pancreatitis reported subsequently [7]. In all the nonfatal cases, of previously reported pancreatitis with this drug, no patient had a prior exposure to alcohol, biliary pathology diagnosed during their hospital course or had a prior history of hypertriglyceridemia [5]. Our patient denied any alcohol consumption and has no previous biliary pathology of note. Furthermore, given our patient’s immunoglobulin G4 levels and the criteria for AIP (autoimmune pancreatitis), IgG4 autoimmune pancreatitis was also ruled out. Our patient presented two weeks after his first dose of brentuximab and the median time to presentation was previously reported as approximately two weeks from the first exposure to brentuximab, with all cases occurring by the third cycle of therapy [5]. Our patient went on to receive further doses of brentuximab without recurrent pancreatitis episodes or worsening in triglyceride levels.

The exact mechanisms involved in hypertriglyceridemia-induced pancreatitis have been previously postulated however still remain to be elucidated. Chylomicrons are believed to cause pancreatic inflammation when triglyceride levels are elevated as they precipitate into the circulation. This impairs capillary bed circulation, causing edema and ischemia to the pancreatic cells, disturbing the pancreatic acinar structure [8-10]. It has been posited that the low levels of CD30 expression in pancreatic cells might be a cause for acute pancreatitis after brentuximab therapy [2]. Our patient developed very severe hypertriglyceridemia after one dose of brentuximab and presented with acute pancreatitis [11,12]. Hence, it is imminent that physicians maintain a high index of suspicion for a rare however life-threatening adverse event with single dose brentuximab therapy to institute emergent and appropriate care for acute pancreatitis.

## Conclusions

Brentuximab vedotin is an antibody-drug conjugate recently approved for the treatment of adult patients with relapsed or refractory Hodgkin lymphoma. An extensive review of the literature does not reveal any other cases of brentuximab-induced hypertriglyceridemia. As the number of clinical studies introducing newer monoclonal antibody therapies increases, it is pertinent for hematologists to be alert about the possibility of such adverse drug reactions and institute immediate medical management.
